# Age-related cognitive effects of the COVID-19 pandemic restrictions and associated mental health changes in Germans

**DOI:** 10.1038/s41598-022-11283-9

**Published:** 2022-05-17

**Authors:** Inga Menze, Patrick Mueller, Notger G. Mueller, Marlen Schmicker

**Affiliations:** 1 German Center for Neurodegenerative Diseases (DZNE), Leipziger Str. 44, 39120 Magdeburg, Germany; 2 Medical Faculty, Otto-Von-Guericke University, Magdeburg, Germany; 3 Center for Behavioral Brain Sciences, Magdeburg, Germany; 4 Research Group Degenerative and Chronic Diseases, Movement, Faculty of Health Sciences Brandenburg, University of Potsdam, Potsdam, Germany

**Keywords:** Psychology, Cognitive neuroscience, Public health

## Abstract

Restrictive means to reduce the spread of the COVID-19 pandemic have not only imposed broad challenges on mental health but might also affect cognitive health. Here we asked how restriction-related changes influence cognitive performance and how age, perceived loneliness, depressiveness and affectedness by restrictions contribute to these effects. 51 Germans completed three assessments of an online based study during the first lockdown in Germany (April 2020), a month later, and during the beginning of the second lockdown (November 2020). Participants completed nine online cognitive tasks of the MyBrainTraining and online questionnaires about their perceived strain and impact on lifestyle factors by the situation (affectedness), perceived loneliness, depressiveness as well as subjective cognitive performance. The results suggested a possible negative impact of depressiveness and affectedness on objective cognitive performance within the course of the lockdown. The younger the participants, the more pronounced these effects were. Loneliness and depressiveness moreover contributed to a worse evaluation of subjective cognition. In addition, especially younger individuals reported increased distress. As important educational and social input has partly been scarce during this pandemic and mental health problems have increased, future research should also assess cognitive long-term consequences.

## Introduction

The Coronavirus disease (COVID-19) was declared a global health emergency in January 2020 (WHO) and has become a still ongoing pandemic with consequences for all areas of human life worldwide. In order to reduce the transmission of the virus, to slow down infection rates and to keep the pandemic at a manageable level, governments around the world have responded to the virus with broad restrictions. As such, the year 2020 was marked by constraints in interpersonal relations, cultural, professional, educational, and economic life. These means to repress the transmission of COVID-19 were shown effective, especially, when they were introduced in early phases of the pandemic^1–4^. However, they also come at a certain cost, which is why research is aiming to understand the disease as well as long-term consequences of the restriction policies since the COVID-19 outbreak. Studies have focused on immediate and long-term health effects as a result of an infection with SARS-CoV-2 as well as mental health changes caused by COVID-19 restrictions^5–9^. The influence of “social distancing” on mental health not only concerns infected people or people with an underlying health condition, but also healthy individuals. E.g. in Germany, restrictions applied for ≈ 83 million habitants while in May 2021 ≈ 3.6 million of those have been infected with SARS-CoV-2^10^ and experienced social isolation during quarantine.

International literature shows that the rate of psychological distress^11–14^ and symptoms of anxiety, depression as well as substance abuse^11^ have increased since the confinements. Li and colleagues^12^ analyzed posts of a common Chinese microblogging webpage and found that after the declaration of the COVID-19 outbreak in China, the amount of posts addressing worries about social risks, health and negative emotions have increased. Another study indicated that the higher the perceived negative impact of the COVID-19 restrictions and the estimated severity of the situation, the higher was the reported stress level of participants, whereby especially women seemed to be affected^13^. Moreover, COVID-19 related restrictions resulted in reduced physical activity, altered sleep patterns, weight gain, and increased alcohol consumption, which affected mental health negatively^5,15–18^. When quarantine was required, its duration, resulting financial losses and insufficient medical care seemed particularly associated with negative effects on mental health^19^.

Kuehner and colleagues^14^ titrated several factors compensating for a negative psychological impact during the COVID-19 pandemic in German participants. A high educational level, high levels of conscientiousness, an internal locus of control, and dispositional optimism were protective regarding psychological well-being. Anxiety, worries, experienced curfews, higher levels of neuroticism, a disposition to ruminate and dispositional pessimism on the other hand, exacerbated the psychological strain. Interestingly it was found, that older participants seemed to be less worried and not as affected by the crisis as younger participants^13,14^. Schlomann and colleagues^20^ argued that older adults bounce back easier from negative effects of a COVID-19 related lockdown as they have a greater life experience and are less demanding with regard to social aspirations. These factors might contribute to a less intense processing of negative information in the context of the pandemic in comparison to younger adults and in turn facilitate resiliency.

Mental health distress due to COVID-19 restriction policies may not be the only challenge as changes of common lifestyle and psychological consequences might also cause implications for cognition. E.g. Depression has been shown to be strongly related to decreasing subjective cognition in older adults during the pandemic^21^. However, research on the consequences on objective cognition is still scarce, although people of all ages are affected to varying degrees by missing cognitive stimulation due to restricted social communication, limitations in educational systems, home office or home schooling. While education has mainly been impacted in younger people, middle-aged adults have primarily been affected in the exertion of professional activities. Besides, both younger and middle-aged adults also faced crucial alterations in exerting leisure time activities. A resulting decrease in cognitive stimulation could also lead to faster cognitive decline in healthy aging. Adverse effects in older adults should therefore be of special interest as cognitive depletion and simultaneous social deprivation could lead to a rapid cognitive decline. As such, higher perceived loneliness negatively affects cognition^22,23^ including attentional control and response inhibition^24^, comprehension, but also satisfaction with life^22^. Besides, social deprivation alone was found to be associated with poorer cognitive status, a faster cognitive decline in people of higher age^25^ and even an increased risk for cardiovascular diseases, depression and Alzheimer’s Disease (AD)^26–32^. Hence, feeling lonely can be considered a risk factor for dementia^30^. Since social and physical distancing aggravated experienced loneliness during this pandemic^33,34^, considering its impact on cognition is extremely important. As older adults are considered to be a high-risk group for COVID-19, physical distancing and thus loneliness may affect this group in particular, assuming that their access to online communication is additionally limited^35^. Nonetheless, younger cohorts need to be taken into consideration, too, as cognitive decline already starts in midlife and neurodegenerative processes begin decades before a diagnosis^36,37^. Early intellectual stimulation is thus important for cognitive development and neuroplasticity^38,39^. Consequently, the COVID-19 related restrictions could depict an additional risk factor for cognitive decline^40,41^.

The goal of this study was to analyze the perceived impact of the restrictions on an individual’s life (here *affectedness*), the depressiveness and loneliness during the COVID-19 related lockdown in Germany and how these factors in turn affected subjective as well as objective cognition across age. For this purpose, an online-based longitudinal study with three assessments was conducted between April 2020 and December 2020. Questionnaires and web-based cognitive tasks were completed by adults of 18 years and older. During the first assessment (April/May 2020), participants faced the first lockdown in Germany. As the lockdown was announced in close temporal proximity to the first assessment, we assumed that restrictions might not have had a huge impact yet. Therefore, mental health proxies were assessed retrospectively and data of the first time point was handled as our reference measurement. In the second assessment period (June 2020), relaxation of restrictions was already given. In the third assessment (November 2020), participants faced a lockdown again. In sum, we were interested in the performance changes of objective cognition and changes in the evaluation of subjective cognition in comparison to the first lockdown or reference measurement respectively. We hypothesized, that higher levels of affectedness by the restrictions as well as higher scores of loneliness and depressiveness would be associated with lower cognitive performance as well as worse subjective evaluation of it and only little improvements in cognitive tasks over time. We further assumed that these relations should vary with age.

## Results

### Description of the sample

The analyzable sample (n = 51, 35 females, *mean*_*age*_ = 43.78, *SD*_*age*_ = 16.56) included 16 young, 17 middle-aged and 18 older participants (Table 1). At none of the assessments participants did report being tested positive with SARS-CoV-2. In all three age groups, participants highly approved of the governmental restrictions and reported high adherence (Supplementary Table S1). Descriptively, older participants showed the highest worries concerning the COVID-19 pandemic. In T2 and T3 the reported worries converged across age groups (Supplementary Table S1).

**Table 1:** Sample characteristics in age groups.

	young	middle-aged	older
*n*	16	17	18
Age (mean, SD)	25.1 (± 2.95)	41.9 (± 9.15)	62.2 (± 5.16)
Age (range)	20–29	32–55	56–74
Females	12	12	11
Quarantined	2	2	0

### Affectedness by restrictions, depressiveness and loneliness

**Figure 1:**
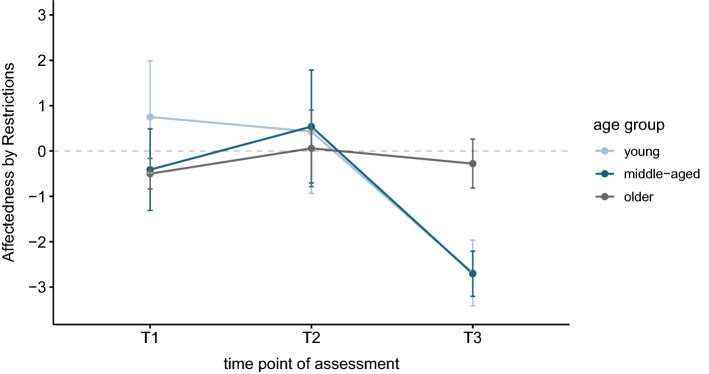
Rated affectedness by the restrictions in the different age groups over time. We used the reported impact of COVID-19 and related restrictions on the communication with friends and family, the physical and psychological health, sleep, nutrition and sporting activities. Participants were asked to rate the impact of COVID-19 on each of these queried domains, ranging from very negative impact (− 2) to very positive impact (+ 2). The affectedness score was then calculated as the sum across items and could range from − 14 to + 14. Negative values represent a negative reported impact, positive values a positive reported impact regarding the affectedness by the restrictions. A value of 0 would indicate no affectedness by COVID-19. Error bars depict 1 standard error.

Levels of affectedness by the restrictions varied between the assessments across age groups. Especially younger and middle-aged participants showed descriptively higher negative affectedness in the third assessment (Fig. 1, see also Supplementary Table S2). We found a positive correlation between affectedness and age in T3 (*r*_*K*_ = 0.29, *p*_*corr*_ = 0.014), which indicated, that with increasing age a less negative impact by the COVID-19-related restrictions was reported.

**Figure 2:**
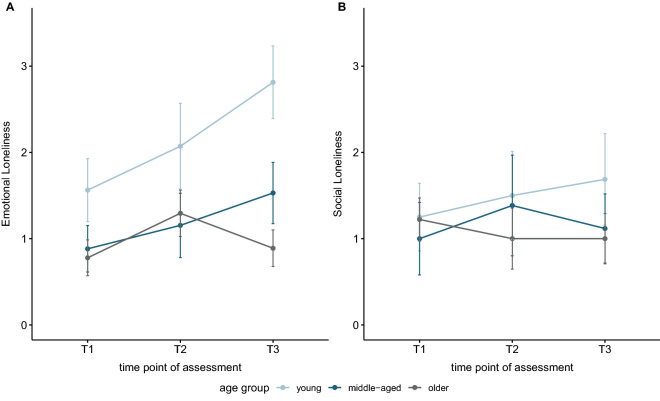
Emotional and social loneliness scores for different age groups across time. Loneliness was assessed by a German version of the 11-items Loneliness Scale by De Jong and Gierveld, which offers a distinction between emotional and social loneliness. Emotional loneliness describes the perceived lack of intimacy in interpersonal relations, whereas social loneliness refers to a lack of a broad social network. Participants gave their answers on a 5-point frequency scale. Emotional loneliness scores can range between 0 and 6, social loneliness scores can range between 0 and 5, with higher scores representing higher perceived emotional or social loneliness respectively. Error bars depict 1 standard error.

Descriptively, an increase in total loneliness across time can be observed, especially in the younger participants (Supplementary Table S3). There was no significant correlation between reported loneliness and age in any assessment. The progression of emotional and social loneliness in the age groups across time are shown in Fig. 2. Regarding emotional loneliness, we found a significant correlation with age in T3 only (*r*_*K*_ = − 0.32, *p*_*corr*_ = 0.007), indicating that emotional loneliness was less prevalent with increasing age. No significant correlations were found between social loneliness and age at any time point.

Reported depressiveness across age-groups is provided in Supplementary Table S3. Correlational analyses showed that there was a negative correlation between age and depressiveness in the third assessment, hinting to higher depressiveness levels in lower age (*r*_*K*_ = − 0.31, *p*_*corr*_ = 0.005). The correlation of depressiveness and age in the second (*r*_*K*_ = − 0.24, *p*_*corr*_ = 0.077) and first assessment (*r*_*K*_ = − 0.23, *p*_*corr*_ = 0.069) did not reach significance after correction.

In order to assess how changes due to restrictions were related to mental health, we furthermore ran correlational analyses between affectedness, loneliness and depressiveness. Concerning affectedness, there was a significant correlation with depressiveness (*r*_*K*_ = − 0.32, *p*_*corr*_ = 0.011) and loneliness (*r*_*K*_ = −0.30, *p*_*corr*_ = 0.023) in T3. These correlations indicated that lower negative affectedness by the restrictions was associated with lower mental health distress only during the second lockdown in Germany. Furthermore, significant positive correlations of depressiveness and loneliness were shown in T2 (*r*_*K*_ = 0.35, *p*_*corr*_ = 0.012) as well as in T3 (*r*_*K*_ = 0.37, *p*_*corr*_ = 0.002).

### Subjective cognition during COVID-19 restrictions in relation to age, affectedness, loneliness and depressiveness

The rating of subjective cognition across the three age groups is depicted in Table 2. There was no significant correlation between age and subjective cognition at any assessment.

**Table 2:** Subjective cognitive rating in age-groups across time.

	T1	T2	T3
Young	69.37 (± 11.10)	62.35 (± 15.73)	58.87 (± 14.06)
Middle-aged	68.75 (± 10.52)	73.84 (± 16.93)	64.06 (± 18.11)
Older	70.22 (± 10.19)	72.90 (± 10.31)	69.24 (± 14.80)

Means and standard deviations in parentheses are reported. Subjective cognition was assessed by 16 items. Participants were asked to indicate how well they coped with the requested skills. They used a visual analog scale ranging from 0 (very poorly) to 100 (very well). The requested skills were derived from dimensions of episodic and implicit memory, executive functions, attention and working memory. The overall mean served as a proxy of general subjective cognitive performance.

However, we found significant correlations between subjective cognition and depressiveness in younger participants in all three assessments (*r*_*K T1*_ = − 0.53, *p*_*corr*_ = 0.022; *r*_*K T2*_ = − 0.75, *p*_*corr*_ ≤ 0.001; *r*_*K T3*_ = − 0.64, *p*_*corr*_ = 0.041). The rating of subjective cognition of middle-aged participants correlated with depressiveness in T1 (*r*_*K*_ = − 0.53, *p*_*corr*_ = 0.018), and with loneliness in T3 (*r*_*K*_ = − 0.51, *p*_*corr*_ = 0.032). For older participants, we found significant correlations of subjective cognition and depressiveness in T2 (*r*_*K*_ = − 0.44, *p*_*corr*_ = 0.018).

The results of the LMEs corroborated these findings (Supplementary Table S4). We found, that expanding the null model, only including age, time and sex as fixed factors, by depressiveness (χ^2^(6) = 29.40, *p* < 0.001) or loneliness (χ^2^(6) = 34.83, *p* < 0.001) significantly enhanced the model fit. The inclusion of affectedness did not enhance the predictive power of the model.

**Figure 3:**
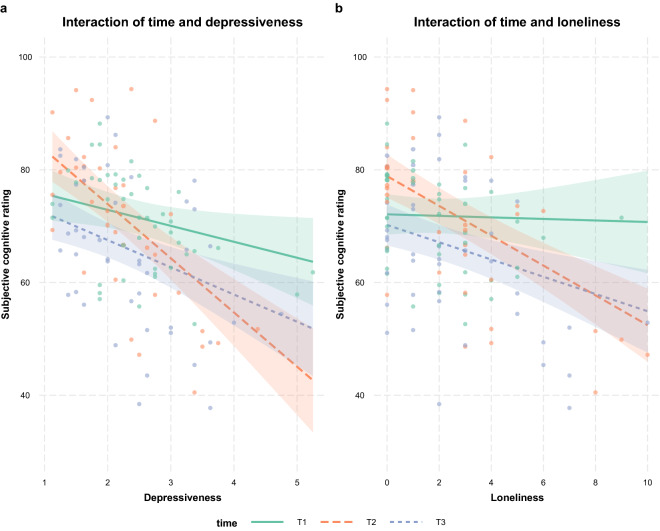
Influence of depressiveness and loneliness on subjective cognitive rating. **(a)** Interactions of depressiveness and time. **(b)** Interaction of loneliness and time. Seven influential data points were removed for the respective analysis.

We found a significant interaction of time x depressiveness in T2 (*β* = − 6.78, *SE* = 1.87, *p* < 0.001, *CI* [− 10.52; − 3.09]), indicating that higher levels of depressiveness in the second measurement led to a more negative rating of subjective cognition compared to T1 (Fig. 3a). In the third measurement, this effect could not be shown. Age did not contribute significantly. Similar interactions were found in the model expanded by loneliness. Here, a significant interaction of time x loneliness was found in T2 (*β* = − 2.51, *SE* = 0.68, *p* < 0.001, *CI* [− 3.85; − 1.16]) as well as in T3 (*β* = − 1.39, *SE* = 0.68, *p* = 0.045, *CI* [− 2.75; − 0.02]), both hinting to a worse rating of subjective cognition with higher loneliness scores compared to T1 (Fig. 3b).

### Objective cognitive performance during COVID-19 restrictions in relation to age, affectedness, loneliness and depressiveness

**Figure 4:**
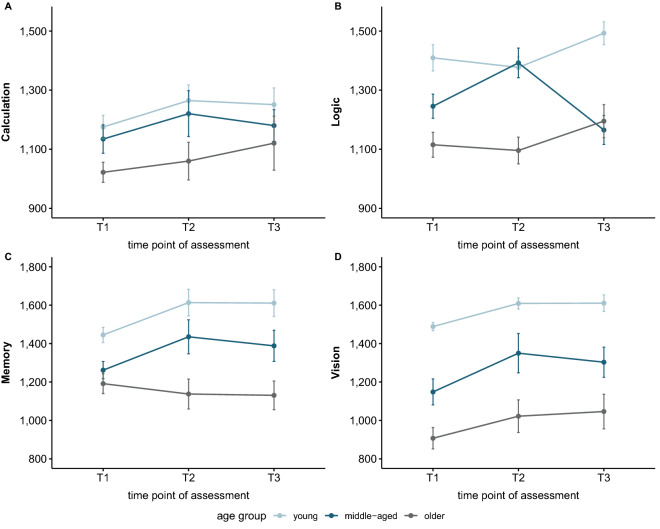
Performance in points within the objective cognitive areas over time. **(a)** Calculation. Assessed with two tasks, in which participants had to solve empty equations or return the right change. **(b)** Logic. Assessed with two tasks, in which participants had to solve a problem deductively and solve a progressive matrices-like task. **(c)** Memory. Assessed with two tasks in which participants conducted a working memory recall and an updating task. **(d)** Vision. Assessed with three tasks, in which participants underwent a feature conjunction visual search, mental rotation and a Stroop-like task.

Objective cognitive performance is reported in scores of 9 cognitive tasks covering the categories memory, calculation, vision and logic which were taken from the applied MyBrainTraining (MBT; neuroCare Group GmbH, https://mybraintraining.com/). Higher scores represent a better performance. The changes of total cognitive performance are reported in Supplementary Table S5. Respective descriptive statistics for all 9 tasks can be retrieved separately from Supplementary Table S6. Figure 4 gives an overview of the performance in MBT’s cognitive areas for the age groups.

#### Calculation

The null model for calculation revealed a significant decrease in performance with regard to increasing age (Table 3). Furthermore, a significant increase in performance was found from first to third assessment. The random intercept accounted for 57.40% of the variance.

**Table 3:** Null models of the objective cognitive performance in tasks of calculation, logic, memory and vision.

Nullmodel	Index	β	SE	95%-CI
Calculation	Sex	70.03	49.80	− 29.59; 169.49
	**Age**	**− 4.51**	**1.65**	**− 7.78; − 1.25**
	T2	40.30	28.07	− 15.15; 96.22
	**T3**	**52.68**	**24.93**	**3.26; 102.10**
	Age x T2	− 2.26	1.67	− 5.57; 1.04
	Age x T3	− 2.03	1.52	− 5.04; 0.98
Logic	sex	− 30.36	42.08	− 114.79; 53.80
	**Age**	**− 6.04**	**1.58**	**− 9.17; − 2.92**
	T2	− 7.25	31.48	− 69.42; 56.09
	T3	− 14.95	27.96	− 70.45; 40.54
	Age x T2	− 0.25	1.95	− 4.15; 3.61
	Age x T3	− 2.61	1.76	− 6.11; 0.89
Memory	Sex	− 2.57	55.87	− 114.36; 108.97
	**Age**	**− 8.21**	**2.12**	**− 12.39; − 4.03**
	**T2**	**125.96**	**41.42**	**44.01; 208.17**
	**T3**	**110.42**	**36.94**	**37.18; 183.65**
	Age x T2	− 3.44	2.58	− 8.55;1.67
	Age x T3	− 3.33	2.34	− 7.95; 1.30
Vision	Sex	41.24	62.86	− 84.64; 166.87
	**Age**	**− 15.39**	**2.14**	**− 19.63; − 11.15**
	**T2**	**101.06**	**32.93**	**36.02; 166.95**
	**T3**	**112.25**	**29.46**	**53.82; 170.67**
	Age x T2	− 1.55	2.02	− 5.55; 2.46
	Age x T3	− 1.19	1.85	− 4.87; 2.49

The following amount of influential data points were excluded for the analyses: Calculation: 1 influential data point; Logic: 4 influential data points; Memory: 3 influential data point; Vision: 3 influential data point. Significant effects are highlighted in bold.

**Figure 5:**
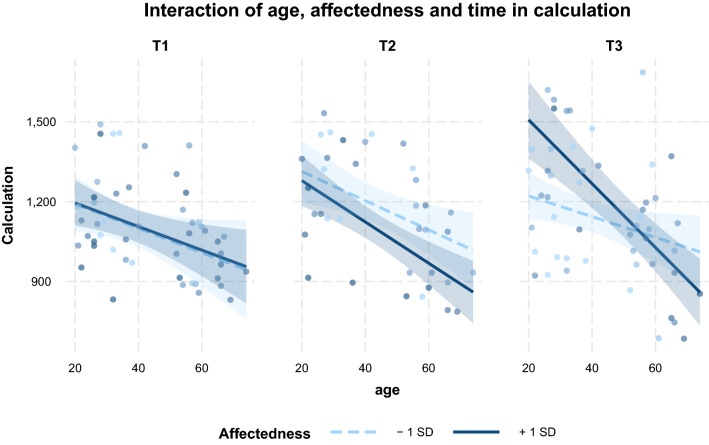
Interaction trend of age, time and affectedness on the calculation task performance. Shaded areas represent 90%-confidence interval.

Loneliness did not contribute significantly to the effect (χ^2^(6) = 9.40, *p* = 0.153). In the full model including affectedness, an interaction trend of age x T3 x affectedness was found (*β* = − 1.17, *SE* = 0.63, *p* = 0.067, *CI* [− 2.43; 0.08], Supplementary Table S7). The trend suggests higher reported affectedness in participants of lower age and furthermore implies that it negatively impacts performance in calculation tasks in these participants. However, this impact remains statistically insignificant (Fig. 5). Overall, adding affectedness as a predictor influenced the model fit of performance in calculation tasks significantly (χ^2^(6) = 14.94, *p* = 0.021). A third full model, complemented by depressiveness also turned out to be superior to the null model (χ^2^(6) = 13.42, *p* = 0.037; Supplementary Table S8), however, the interaction terms of age x time x depressiveness did not yield significance. Furthermore, the model fit was slightly inferior to the full model of affectedness (AIC_Affectedness_ = 1790.1, AIC_Depressiveness_ = 1791.6).

#### Logic

The null model revealed a significant effect of age on performance in logic tasks (Table 3; Supplementary Fig. S9). The random intercept accounted for 37.66% of the variance. Neither the inclusion of affectedness, loneliness nor depressiveness explained significantly more variance in the performance in logic tasks across age and time.

#### Memory

We found a significant age effect in the null model, hinting to a decrease in performance with increasing age (Table 3). Furthermore, performance gains from first to second assessment, and first to third assessment became significant. The random intercept accounted for 38.55% of the variance.

**Figure 6:**
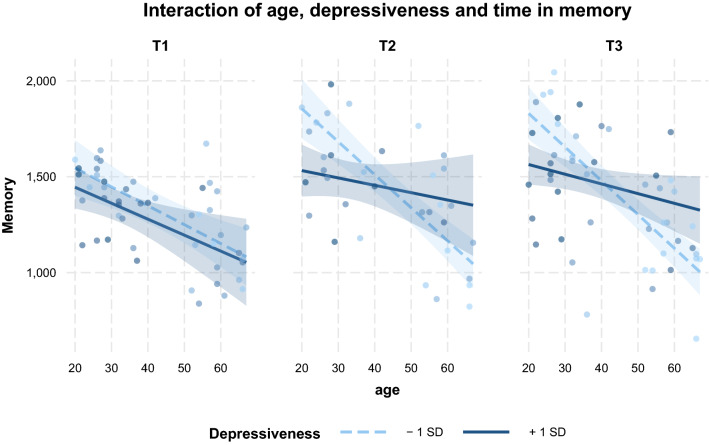
Interaction between age, depressiveness and time on memory task performance. Shaded areas represent 90%-confidence interval.

Neither the full model including affectedness, nor the full model including loneliness explained further variance. Depressiveness, however, contributed significantly to performance in memory tasks (χ2(6) = 16.90, *p* = 0.010). There was a significant interaction between age x T3 x depressiveness (*β* = 5.85, *SE* = 2.91, *p* = 0.047, *CI* [0.10; 11.62]; Supplementary Table S10), indicating that depressiveness levels had a different impact on performance changes from T1 to T3 with regard to age (Fig. 6). The interaction implies that the younger the participants, the stronger the detrimental effect of depressiveness on improvements in memory task performance.

#### Vision

The null model revealed a lower performance in higher age (Table 3; Supplementary Fig. S11). Furthermore, a significant increase in performance was found from first to second and first to third assessment. The random intercept accounted for 61.95% of the variance. Neither the inclusion of affectedness, loneliness nor depressiveness explained significantly more variance in the performance in vision tasks across age and time.

## Discussion

This study aimed to analyze the COVID-19 restriction-related longitudinal changes in mental health and their effects on subjective as well as objective cognitive performance. Our findings give a hint to a negative influence of psychological distress on cognitive performance as a consequence of restrictions. Thereby, restrictions seemed to affect younger and middle-aged adults more than older participants and resulted in greater psychological distress, which negatively affected subjective cognitive performance and appeared to attenuate improvements in calculation tasks as well as memory tasks over time. To understand the contributions of COVID-19 restriction-related changes on subjective as well as objective cognition across age, we have to shed light on ratings of affectedness, related mental health changes and their particular influences on cognition.

### Affectedness by restrictions, loneliness and depressiveness

Our analysis revealed that the reported affectedness by the restrictions decreased with increasing age. As such, the younger the participants, the more negative impact they reported by the restrictions in T3. This might be related to stronger, prolonged changes in their everyday-life, due to altered educational or professional situations^13,42^. In fact, it was reported that middle-aged individuals reported a higher decrease in satisfaction with life and social contacts in relation to a COVID-19 lockdown, as compared to older individuals^20^. Moreover, especially people of younger age appear to be worried by economic consequences and the unpredictability of the course of the pandemic^13^. The feeling of uncertainty towards a situation appears to be an influential factor for developing mental health problems^43^ and feeling lonely^44^. Younger people might also be more affected by social distancing measures due to the perceived relevance of social activities^45^, which makes them more vulnerable to feeling lonely^34^. The importance of social networks seems to be closer related to younger age, whereas older individuals rather have smaller social networks, with more intimate contacts^33,46,47^. In turn, it is assumed that perceived loneliness in older adults is not necessarily dependent on the quantity of contacts but more heavily influenced by the quality of contacts^48,49^. In line with that, we found that the younger the participants the more emotional loneliness was reported in T3. Similar results have been reported multiple times^13,34,42,50,51^. As such, older age is not necessarily a risk factor for feeling lonely, but rather affects those, who are immobile or functionally limited^47,51^. In a diary-based survey in the first four weeks of restrictions in Germany, Buecker and colleagues^33^ found that perceived daily loneliness increased within the first two weeks, but then decreased again. This effect was stronger with increasing age. Moreover, a qualitative study found that symptoms of feeling lonely or depressed didn’t necessarily aggravate as a result of pandemic-related restrictions in older adults who are already prone to or experience loneliness and depression^52^. Regarding our sample of older adults who appear to be familiar with digital media, as they participated in this online study, we additionally need to point out that they might not have been as “isolated” as their peers, who do not have access to (social) media. In fact, the usage of video calls in Germany in the first quarter of 2020 had increased by 9% compared to the previous year^53^. In contrast, the increase in video calls from 2018 to 2019 was only 2%^54^. Even though we didn’t find particular negative effects in older adults here, further research is needed to assess the situation of more vulnerable groups or groups at risk, like nursing home residents^29^.

The observed higher depressiveness in lower age during the second lockdown (T3) might be related to similar factors due to COVID-19 restrictions but could also be influenced by external changes which we were not able to control for. As the second lockdown coincided with winter season, there might have been an increased risk for winter seasonal affective disorder. Yet, depressiveness levels overall were rather low to moderate in our sample. How more vulnerable individuals are affected in the course of this pandemic, hence, still needs to be addressed more thoroughly, especially since these groups are prone to aggravation of their symptoms^42,50,55^.

It should be emphasized that also positive effects of a lockdown were reported in an Indian study, which concerned improvements in relationships to other people, such as family members but also colleagues or neighbors^56^. These effects, however, occurred with a concurrent increase of negative emotions. A Scottish survey reported positive changes due to the lockdown, too, which were however associated with specific sociodemographic characteristics^57^. Those included especially younger age groups, females, those who were married or living with their partner, those who were employed and those in good health. They reported experiencing positive changes e.g. in interpersonal relations, more time for enjoyable leisure time activities and time for contemplation.

### Subjective cognition

We found evidence for a poorer evaluation of subjective cognition during the COVID-19 related lockdown, which was related to depressiveness and loneliness. Younger adults reported a lower subjective cognitive performance over time and this decline was associated with higher depressiveness. In participants older than 30 years the decreasing subjective cognition rating came along with higher depressiveness in the first and higher perceived loneliness in the third measurement. In older participants, higher depressiveness resulted in lower subjective cognition ratings in the second measurement. People with depression typically complain about concentration and memory problems^58^ and a depressed mood affects attention objectively as well as subjectively^59^. Also loneliness is thought to mediate the relationship between social isolation and subjective cognitive impairment^60^. Consistent with our finding, Fiorenzato and colleagues^61^ furthermore reported that changes in subjective cognition of younger people were present during the confinements in Italy, too. Comparable to our result, higher prevalence of mental health problems, such as depression or sleep disorders, were related to a decrease in reported subjective cognition.

### Objective Cognitive performance

Overall, data from cognitive tasks showed that participants slightly increased their performance across time, which speaks for general training effects independent of age. These training effects occurred mainly from T1 to T2 and then stagnated from T2 to T3. LMEs revealed that slopes from T1 to T2 are similar to slopes from T1 to T3. While the time between the first two measurements was characterized by loosening of restrictions, the period before the third time point was marked by strict social distancing rules and the announcement of the 2nd lockdown in November. Furthermore, training effects might have vanished due to the large time interval.

In detail, objective cognitive performance was measured in 9 tasks which were retrieved from MBT’s four categories of cognitive tasks, namely calculation, memory, vision and logic. Results show that perceived depressiveness impedes performance improvements in memory tasks. The younger the participants, the stronger the detrimental effects of depressiveness were on task performance. In contrast to our expectations, we only observed a trend towards negative affectedness impacting calculation task performance in younger age, but this effect did not reach statistical significance. For logic task performance the LME revealed a significant age effect, but performance changes did not yield significance. Finally, all participants slightly increased their performance in vision tasks over time and neither mental health factors nor affectedness modulated this ability.

In fact, increased psychological distress can lead to hyperarousal and increased anxiety, which in turn negatively impact cognitive functions^62^. Taken the findings together, it seems that middle-aged and younger individuals might be particularly vulnerable to the broad changes in the context of the pandemic, although the impact on objective cognitive performance was limited. In an online-based study that assessed the cognitive functions across 5 time points during different phases of COVID-19 related restrictions, Ingram, Hand and Maciejewski similarly demonstrated the negative impact of social isolation on cognitive performance^63^. This impact was shown across age and different cognitive tasks such as decision making or attentional performance. However, when restrictions were loosened, the authors observed performance improvements. As we also found higher reported affectedness, and loneliness in the younger and middle-aged participants, we similarly assume that mental health distress due to restrictions might have had an impact on the cognitive performance. As such, absence of task improvements could be a consequence of restrictions, lower cognitive stimulation during home-office and social isolation. However, our sample is too small to draw definite conclusions and our results should hence be interpreted carefully. Still, the effects of COVID-19 restriction-related changes on cognition in people suffering from social distancing and alterations in professional life have to be a focus in future research. Furthermore, the question should be addressed whether some cognitive abilities are less impacted by psychologically mediated pandemic effects of social isolation.

On the one hand, the results additionally might imply a higher resilience in German older adults to the COVID-19 related restrictions and their consequences, not only with regard to the evaluation of and coping with the situation, but also with regard to the impact on cognitive abilities. Thus, age could be assumed a protective factor in light of exceptional situations, such as the COVID-19 pandemic. Older adults might be more capable to adapt to such extraordinary situations. Similarly, a study in earthquake survivors found that increasing age was linked to higher resilience^64^. On the other hand, restrictions were also shown to decrease cognition in older people at high risk to develop dementia. An analysis of the progression of Mini-Mental-State scores in the period before the restrictions of the first pandemic wave and within that wave revealed an acceleration of cognitive decline in a sample of cognitively impaired individuals^65^. A similar effect was demonstrated in people in prodromal phases like mild cognitive impairment^66^, although these effects varied on an individual level since participants who engaged in leisure time activities, talked to others on the phone or conducted cognitive training at home showed lower cognitive decline. Such activities are associated with establishing a strong cognitive reserve, which is protective against neurodegenerative diseases^67–71^.

### Limitations

Several limitations of the current study must be considered. First, no appropriate baseline data without influences of COVID-19 was available for comparison, which is a relatively common issue in COVID-19 research due to the quick series of events making it difficult to conduct studies in a timely manner and it has been reported in other studies, as well (e.g.^20,66^). Yet, we assumed that due to the close proximity of the first assessment to the start of the first lockdown, there should not be a considerable impact on subjective and objective cognitive functioning or on depressiveness and loneliness. Furthermore, the affectedness score, which was close to 0 across age groups in this first assessment, resembles that the experienced restrictions were not rated to have a considerable impact on participants. These reasons made us believe that although there is a time mismatch between the measurement, the values assessed at T1 can serve as a reference. Nevertheless, we emphasize to regard the results with caution. Second, we note that MBT is not a validated test battery but a cognitive training tool. We used it, however, for its low-threshold availability and its motivating, ludic design, which was assumed to increase participants’ adherence. Third, due to a relatively high drop-out rate, the sample size was critically reduced which can be traced to the lengthy assessment, resulting in lower motivation and engagement^72^. Hence, we would like to point out that the data should be regarded as rather exploratory and, above all, interpreted with caution. Fourth, our sample might not be perfectly representative to draw a generalization. As participation required access to the internet, a selection bias can be assumed, especially for the older adults. Higher educated people with media competence could have been attracted by the study. In contrast, older people in residential or long-term care facilities have been imposed by home confinement and might suffer more severely from missing interaction with spouses, families and friends outside their residential context. This reduction of social contacts could lead to social deprivation and results in cognitive disadvantages after longer periods of persistent loneliness^28,73,74^. Accordingly, a study by O’Caoimh and colleagues hinted to a stronger effect of social deprivation on cognitively impaired care home residents as assessed by caregivers in contrast to cognitively unimpaired residents^75^. Moreover, they found that the visitation restrictions had a negative impact on the caregivers’ well-being, too. We further need to point out, that conformity and approval of the imposed restrictions was generally high in our sample. A comparison between highly adhering and low adhering individuals is strongly encouraged. Fifth, our sample comprised predominantly women and only people from age 18 on. Nevertheless, younger children and young adolescents can be considered particularly vulnerable to the situation due to profound developmental tasks^46^. Hence, final inferences for the general German population should be made with caution.

As we discussed above, our findings are consistent with results of other studies outside Germany. However, restrictions and their onset were not identical across countries. Cultural differences could also influence the coping with the containment means and thus also mental health and cognition. For example perceived loneliness differs between European countries^76^, which could cause different effects across countries. Further longitudinal, international, and cross-national research in this field is needed to paint a more holistic picture.

## Conclusion

To the best of our knowledge, this is one of the first longitudinal studies investigating the consequences of psychological distress on cognitive abilities in the context of the COVID-19 pandemic. Apart from the high relevance of examining psychological short- and long-term consequences, it points to the necessity to set the focus on cognitive health and keep consequences of the pandemic in mind holistically in order to establish strategies to decrease distress caused by future pandemic related restrictions—not only for the sake of mental health but also for the sake of cognitive health. As such, it was recently argued, that a low incidence, transnational approach could allow for a reduced need of restrictions while counteracting the disadvantages of high incidence rates such as economic consequences or overburdening health care and educational systems^77^. Our findings imply higher negative evaluation of changes due to restrictions and higher levels of depressiveness, particularly in younger age cohorts. Those seemed to have a negative impact on objective cognitive performance, which was however limited to certain tasks. Independent of age, increased loneliness and depressiveness appeared to affect subjective cognition. Accordingly, awareness should be raised to possible cognitive consequences of COVID-19 related restrictions particularly for cognitively vulnerable groups, e.g. mild cognitively impaired or subjectively cognitive declined patients.

## Materials and methods

### Sample

**Figure 7:**
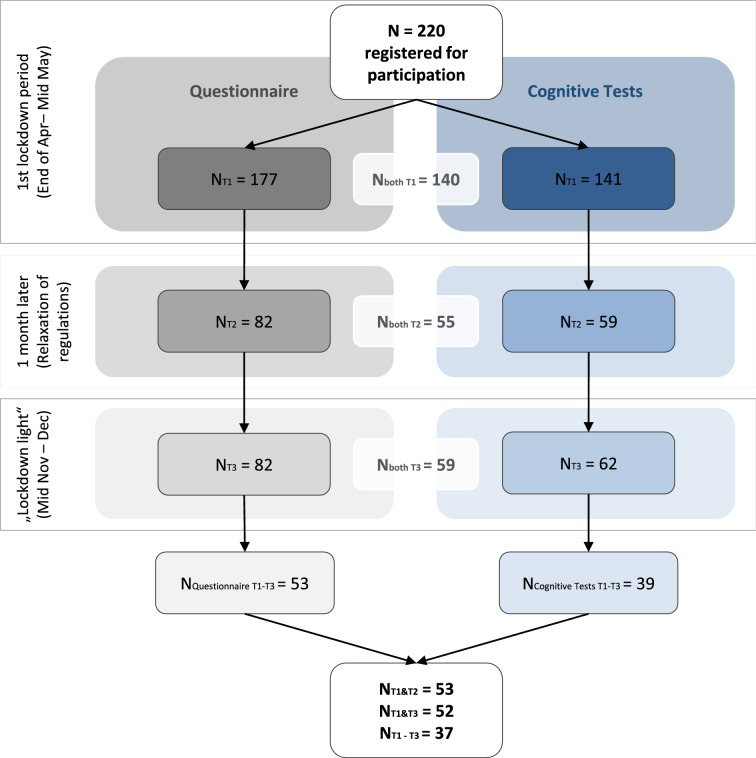
Flow Chart of Participants and dropouts over the three assessments for the questionnaire on SoSci Survey as well as the online-based cognitive tasks with MyBrainTraining. From the 220 registered participants, 196 took part in at least one measurement time point and completed at least one task. As the questionnaire and the cognitive tasks could be completed independently, the number of participants differs for the questionnaire and cognitive tasks across time points. As such, 140 participants in the first assessment finished the questionnaire as well as the cognitive tasks (94 females). The age of this initial sample ranged from 20 to 77 years (*mean*_*age*_ = 43.53, *SD*_*age*_ = 15.94). In the second assessment 55 participants completed both the questionnaire and the cognitive tasks (40 females, *mean*_*age*_ = 44.05, *SD*_*age*_ = 17.05). In the third assessment, 59 participants finished both tasks (40 females, *mean*_*age*_ = 44.90, *SD*_*age*_ = 16.60). Overall, 37 participants completed all three assessments. The analyzed sample of 51 participants who finished both assessments in T1 and T3 included 12 students, 11 retirees and 28 employed participants.

German-speaking people aged 18 years and older could participate in the online based study (Fig. 7). There were no further exclusion criteria. Recruitment was realized via social media, the institute’s webpage, and local newspaper advertisement. Participants gave their informed consent in accordance with the Declaration of Helsinki. The study was approved by the ethics committee of the University of Magdeburg (Germany). Participants received no financial compensation but were offered feedback about their cognitive performance after the third assessment.

### Procedure

**Figure 8:**
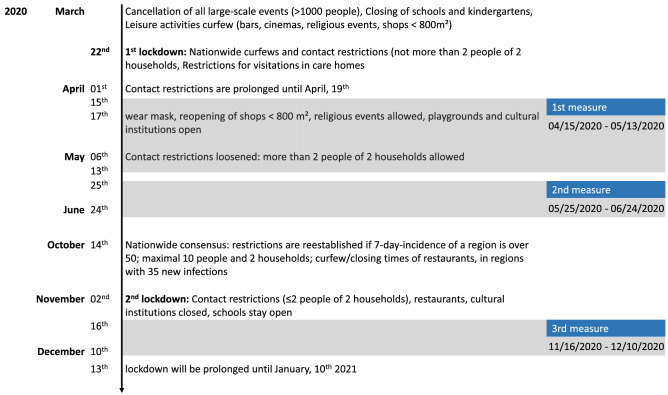
Timeline of COVID-19 related restrictions in Germany. During the first assessment, the first lockdown occurred in Germany, first relaxations of regulations were made on May, 8th 2020. During the second assessment regulations were further loosened. The third assessment coincided with the second lockdown in Germany.

After registration, participants were sent a link for the online questionnaire and online cognitive assessment. The first assessment took place between the end of April 2020 and mid-May 2020 (Fig. 8). The second assessment followed a month after. The third assessment was between mid-November 2020 and the beginning of December 2020.

### Online task material

#### Cognitive tasks

The cognitive assessment was realized with MyBrainTraining (MBT; neuroCare Group GmbH, https://mybraintraining.com/). MBT is a cognitive remediation training application that is available web-based as well as app-based for iOS or Android. In total, it contains 30 tasks, which are divided into four task categories according to MBT, namely in the area of “calculation”, “logic”, “memory” and “vision”. Difficulty within tasks is adapted automatically according to the participant’s performance. Overall performance is presented in points based on difficulty, correct answers within a task and time needed to finish the task. For the current study, 9 different tasks of the cognitive areas according to MBT task categories were chosen for the cognitive assessment and conducted by each participant (Table 4).

**Table ﻿4:** Overview of the tasks of MyBrainTraining.

Name of task	Task instruction	Cognitive area
Empty equations	Fill in the empty spaces in an equation with the missing numbers	Calculation
Give the right change	Return the difference between the price and the given amount with as few coins as possible	Calculation
Evolution revolution	Recognize the offspring of the two parent-beings by its attributes; correct offspring only carries attributes of its parents	Logic
Which is the heaviest?	Detect the heaviest object on the scale in the image	Logic
Picture sequence	Remember the order of appearing objects and report them in the same order in recall	Memory
Fruit basket	Remember the correct number of fruits which are put in a basket, occasionally some fruits leave the basket again. Report the correct number of the queried fruits in the basket	Memory
Match the pairs	Recognize all cards in the array that form pairs as quickly as possible	Vision
Stroop test	Report the colors in which the shown words are displayed	Vision
Point of view	Geometric objects are depicted, decide from which angle the image is viewed	Vision

The tasks and their order were held constant over all participants for all three time points. The selection of the 9 tasks was based on the goal to assess cognitive performance across all areas of MBTs task categories, namely calculation, logic, memory and vision. In the column task instruction, the task’s demands are briefly described. In the column cognitive area, the allocation of the tasks to MBTs task categories is reported. Depending on the participant’s choice, the cognitive tasks were executed either in the web-browser or via the app on her*his smartphone. Participants were instructed to complete the tasks in a distraction-free environment and to focus on the tasks.

#### Online based questionnaire

The questionnaire was conducted via SoSci Survey^78^ and was accessible online on www.soscisurvey.de. The questionnaires of all three assessments consisted of a question catalog, which was part of a bigger study project. The first questionnaire was the longest, as we assessed further traits here as well (30 to 45 min). The questionnaires of the second and third assessment were shorter and took between 15 and 30 min. The focus of the current study was the influence of perceived affectedness by the restrictions, loneliness and depressiveness on subjective as well as objective cognition.

To assess the psychological strain and impact on lifestyle factors caused by the restrictions, which we here subsume under the term “affectedness”, we aggregated an affectedness score. We used the reported impact of COVID-19 and related restrictions on the communication with friends and family, the physical and psychological health, sleep quality, nutrition and sporting activities (“Please rate, how the Corona-Virus impacted the following domains?”). All these factors contribute to a healthy lifestyle and are in turn assumed to be protective of cognitive health^38,71^. On a 5-point polarity profile, participants were asked to rate the impact of COVID-19 on each of the 7 queried aspects, ranging from very negative impact (− 2) to very positive impact (+ 2). The middle category corresponded to no change. The affectedness score was then calculated as the sum across items and could range from − 14 to + 14. Negative values represent a negative reported impact, positive values a positive reported impact regarding the affectedness by the restrictions. A value of 0 would indicate no affectedness by COVID-19.

Loneliness was assessed by a German version of the 11-items Loneliness Scale by De Jong and Gierveld^79,80^, with which emotional as well as social loneliness can be assessed. Emotional loneliness describes the perceived lack of intimacy in interpersonal relations, whereas social loneliness refers to a lack of a broad social network. Participants gave their answers on a 5-point frequency scale, rating how well the statements applied to them. The total loneliness score ranges from 0 to 11 with higher scores indicating higher perceived loneliness. In the first assessment, participants rated their loneliness in general, i.e. before the restrictions. In the second and third assessment, they were asked to rate how well the statements currently applied to them.

Depressiveness was assessed with the German scale “depressiveness in non-clinical contexts”^81^. The scale is based on the depression model by Beck and encompasses 8 items. In the first assessment, participants were asked to evaluate their general depressiveness before the restrictions, whereas in the second and third assessment the evaluation was related to the ongoing restriction period. Answers were given on a 7-point frequency scale. Depressiveness scores range from 1 to 7, whereby higher depressiveness is associated with a higher score.

Moreover, participants were asked to rate their subjective cognitive performance. Subjective cognition was assessed by 16 items, partly adapted from common assessments of the Questionnaire for Experiences of Attention Deficits^82^, the 20-item version of the Everyday Memory Questionnaire^83^ and the Dysexecutive Questionnaire^84^. Participants were asked to indicate how well they coped with the requested skills. They used a visual analog scale ranging from 0 (very poorly) to 100 (very well). The requested skills were derived from dimensions such as episodic and implicit memory, executive functions, attention and working memory (see Supplementary Table S12). The overall mean served as a proxy of general subjective cognitive performance.

### Data analyses

Changes in cognitive performance over time were the primary outcome of the study. We used the scores of MBT for the analysis, as they take the difficulty level, the solving time as well as the ratio of correct answers into account. Performance was separately analyzed for the mean performance in MBT’s cognitive task areas calculation, logic, memory and vision (Table 4).

The main objective was to analyze, how performance changes over time in the cognitive areas as well as in subjective cognitive performance were affected by age, the reported affectedness by the restrictions, perceived loneliness and depressiveness. For this purpose, we first report the descriptive statistics of the relevant variables of affectedness by the restrictions, loneliness and depressiveness. For reasons of clarity, we report these statistics separately for the different age groups. Given the age distribution in our sample, we determined three age groups that broadly defined younger participants, aged below 31 years, middle-aged participants (31 to 55 years) and older participants, aged older than 55 years. It needs to be stressed, that this distinction was made for visualization and descriptive purposes. We then ran correlational analysis to analyze the relationship between age and affectedness by the restrictions, depressiveness as well as loneliness across the assessments. Correlational analyses were conducted via Pearson if normal distribution was given, or via Kendall in case of not normally distributed data or ordinal data. Correction for multiple comparisons was realized using the Bonferroni method.

To answer the main research question, whether affectedness by the restrictions, loneliness and depressiveness are influencing the course of cognitive performance, we analyzed their impact on subjective cognitive performance as well as objective cognitive performance in the four cognitive areas by using linear mixed effects models (LME). Age was hereby used as a continuous variable to acknowledge its variance. We also used age as a centered variable around the mean, to give more meaning to the intercepts. As LMEs are robust against missing data, we opted to analyze data of participants, who fully completed the first and the third assessment of the study and allowed missing data in subjective or objective cognitive performance only in T2. Among the remaining 52 participants, data on the outcome variables in T2 (4 young, 7 middle-aged, 3 elderly) was missing for 14 participants. One further participant had to be excluded, as she*he completed the cognitive tasks but didn’t provide data on the predictive variables of the questionnaire in T2. The analyzed sample therefore consisted of 51 participants.

LMEs were calculated in R version 4.0.2 using RStudio version 1.3.1073^85^ via the packages lme4^86^, lmerTest^87^ and psych^88^. Figures were created with the packages ggplot2^89^ and interact^90^. Preconditions of linearity and homoscedasticity of residuals were checked via Shapiro–Wilk tests and by visual inspection of the QQ-plots. Influential data points were determined by Cook’s distance. To compare models with each other, the influential data points which significantly changed the slopes across all models were excluded from further analysis.

First, random intercept null models were calculated for subjective cognition and for each cognitive area, respectively. These null models only included the fixed factors sex (control variable; female as reference category), age and time. Time was modeled as a three-level factor. We opted for this solution, as we were not analyzing a sole time-dependent effect, but rather a restriction-related effect. As such, in the first (T1) and third assessment (T3), participants faced a lockdown. In the second assessment period (T2), restrictive means were already loosened. However, since we assumed that restrictions in T1 might not already have had a huge impact, mental health proxies were accordingly assessed retrospectively and the first measurement was handled as our reference measurement. We were therefore interested in the performance changes in comparison to the reference. Each null model included an interaction term for age and time, to test whether there are age specific performance changes over time. Participants were treated as random factors to account for different performance at the reference measurement. Random intercept full models were calculated which extended the interaction term of the respective null models by the additional predictors affectedness by restrictions, loneliness or depressiveness. We were especially interested in the three-fold interactions. After each extension, the full model and the null model were tested against each other via Likelihood-ratio tests. The Alpha-level for all analyses was set to *α* = 0.05. Furthermore, 95% confidence intervals were calculated.

## Supplementary Information

Supplementary Information.

## Data Availability

The datasets generated and/or analyzed during the current study are available from the corresponding author IM (inga.menze@dzne.de) or from MS (marlen.schmicker@dzne.de) on reasonable request.
