# Supplementary material for: Age-related cognitive effects of the COVID-19 pandemic restrictions and associated mental health changes in Germans
Source: Sci Rep. 2022 May 17;12:8172. doi: 10.1038/s41598-022-11283-9 (PMC9112263; doi:10.1038/s41598-022-11283-9)
Supplement: Supplementary file 1 — Supplementary Information. [file 41598_2022_11283_MOESM1_ESM.pdf]

Supplements

Supplementary Table S1. *Approval, adherence to the German government restrictions and worries concerning COVID-19 in the age groups across time.*

		T1	T2	T3
**Approval**	**young**	6.0	5.5	5.0
	**middle-aged**	5.0	5.0	5.0
	**older**	5.0	5.0	5.0
**Adherence**	**young**	5.0	5.0	5.0
	**middle-aged**	5.0	5.0	6.0
	**older**	6.0	5.0	5.5
**Worries**	**young**	3.0	4.5	3.5
	**middle-aged**	4.0	4.0	3.0
	**older**	5.0	4.0	2.5

Annotations. Participants were asked to report their approval, adherence and worries concerning the governmental restrictions on a 6-point Likert-scale. Median is reported. 1 = no approval/adherence/worries; 6 = high approval/adherence/worries.

Supplementary Table S2. *Affectedness by the restrictions for the respective age groups.*

	T1	T2	T3
**young**	0.75 (±4.95)	0.43 (±5.09)	-2.69 (±2.89)
**middle-aged**	-0.41 (±3.71)	0.54 (±4.48)	-2.71 (±2.05)
**older**	-0.50 (±1.42)	0.06 (±3.47)	-0.28 (±2.30)

Annotations. Means and standard deviations in parentheses are reported. The affectedness score could range from -14 to +14. Negative values represent a negative reported impact, positive values a positive reported impact regarding the affectedness by the restrictions. A value of 0 would indicate no affectedness by COVID-19.A value of 0 would indicate no affectedness by COVID-19.

Supplementary Table S3. *Depressiveness and total loneliness for the age groups across the three measurement time points.*

		Total Loneliness	Depressiveness
T1	young	2.82 (±2.83)	2.91 (±1.09)
	middle-aged	1.88 (±2.00)	2.58 (±0.64)
	older	2.00 (±1.37)	2.27 (±0.81)
T2	young	3.57 (±3.52)	2.72 (±1.02)
	middle-aged	2.54 (±3.07)	2.34 (±0.71)
	older	2.29 (±1.86)	2.00 (±0.85)
T3	young	4.50 (±3.54)	3.08 (±1.27)
	middle-aged	2.65 (±2.67)	2.36 (±0.84)
	older	1.89 (±1.81)	1.95 (±0.72)

Annotations. Means and standard deviations in parentheses are reported. Depressiveness ranged from 1-7, higher values indicating higher depressiveness. Loneliness ranged from 0-11, with higher values indicating higher loneliness.

Supplementary Table S4. *LME models for subjective cognitive performance. Null model, full model extended by depressiveness, full model extended by loneliness.*

Factor	Null model			Full model depressiveness			Full model loneliness		
	**β**	**SE**	**95%-CI**	**β**	**SE**	**95%-CI**	**β**	**SE**	**95%-CI**
sex	-3.65	3.34	-10.35; 3.04	-2.44	2.85	-8.18; 3.22	-3.84	2.92	-9.70; 2.00
age	-0.01	0.11	-0.24; 0.21	-0.14	0.25	-0.64; 0.37	-0.01	0.12	-0.25; 0.23
T2	-0.20	1.51	-3.21; 2.81	14.60	4.64	5.43; 23.86	6.79	1.98	-0.25; 0.23
T3	-5.54	1.43	-8.36; -2.71	-1.36	4.53	-10.37; 7.61	-1.89	1.89	-5.65; 1.85
age x T2	0.11	0.09	-0.08; 0.29	-0.13	0.30	-0.73; 0.45	0.23	0.12	-0.02; 0.47
age x T3	0.14	0.09	-0.04; 0.32	0.12	0.28	-0.44; 0.68	0.05	0.12	-0.19; 0.29
depressiveness/loneliness				-2.83	1.53	-5.94; 0.22	-0.14	-0.63	-1.44; 1.14
age x *depressiveness/loneliness*				0.04	0.09	-0.16; 0.23	-0.00	0.04	-0.08; 0.08
depressiveness/loneliness x T2				-6.78	1.87	-10.522; -3.09	-2.51	0.68	-3.85; -1.16
depressiveness/loneliness x T3				-2.00	1.84	-5.64; 1.65	-1.39	-0.68	-2.75; -0.02
age x T2 x *depressiveness/loneliness*				0.08	0.12	-0.16; 0.33	-0.05	0.04	-0.133; 0.04
age x T3 x *depressiveness/loneliness*				-0.01	0.12	-0.24; 0.22	-0.02	0.04	-0.07; 0.10

Supplementary Table S5. *Total Points overall in MBT for the respective age groups.*

	**T1**	**T2**	**T3**
**young**	1391.72 (±77.78)	1481.92 (±74.26)	1504.50 (±115.90)
**middle-aged**	1192.02 (±153.83)	1349.60 (±187.91)	1264.00 (±224.18)
**older**	1042.02 (±151.18)	1072.46 (±220.54)	1114.59 (±296.91)

Annotations. Means and standard deviations in parentheses are reported.

Supplementary Table S6. *Mean of Points for the respective age groups across the three assessments.*

	T1			T2			T3		
	**young**	**middle-aged**	**older**	**young**	**middle-aged**	**older**	**young**	**middle-aged**	**older**
**Empty Equations**	1180.63 (±161.16)	1051.76 (±129.41)	970.40 (±154.00)	1125.08 (±148.87)	1023.90 (±238.68)	972.80 (±174.12)	1099.63 (±148.20)	1043.77 (±166.02)	1026.33 (±353.78)
**Give the Right Change**	1169.81 (±187.05)	1217.24 (±286.18)	1073.33 (±210.00)	1404.25 (±381.90)	1416.70 (±397.78)	1146.93 (±381.79)	1402.19 (±376.98)	1316.24 (±392.25)	1214.72 (±474.53)
**Evolution Revolution**	1463.06 (±233.72)	1247.47 (±228.78)	1151.78 (±182.04)	1357.83 (±206.01)	1313.20 (±218.76)	1108.87 (±187.27)	1564.88 (±186.58)	1164.94 (±225.57)	1230.11 (±238.09)
**Which is the Heaviest?**	1355.50 (±237.96)	1243.53 (±203.51)	1078.33 (±249.26)	1395.92 (±167.04)	1471.40 (±186.46)	1082.60 (±228.45)	1420.44 (±224.58)	1164.94 (±254.42)	1160.00 (±329.85)
**Point of View**	1072.81 (±202.09)	813.18 (±437.26)	552.28 (±407.05)	1199.92 (±238.64)	1064.20 (±428.70)	822.13 (±314.29)	1292.63 (±279.66)	999.00 (±494.77)	758.89 (±461.73)
**Picture Sequence**	1262.88 (±153.67)	1050.71 (±206.45)	983.78 (±245.70)	1375.25 (±282.91)	1313.60 (±280.05)	1017.93 (±333.73)	1361.31 (±332.99)	1163.53 (±337.88)	953.33 (±330.88)
**Fruit Basket**	1627.56 (±258.12)	1472.71 (±290.31)	1399.11 (±276.32)	1851.67 (±384.63)	1557.20 (±335.30)	1257.07 (±389.52)	1860.44 (±415.86)	1613.12 (±522.86)	1307.94 (±467.41)
**Match the Pairs**	1733.88 (±112.69)	1578.18 (±142.41)	1414.50 (±203.38)	1824.50 (±233.40)	1696.50 (±302.14)	1391.33 (±246.64)	1796.75 (±188.47)	1532.24 (±298.20)	1337.00 (±304.48)
**Stroop Test**	1659.38 (±152.95)	1053.41 (±508.31)	754.61 (±329.77)	1802.83 (±166.99)	1289.70 (±456.86)	852.47 (±546.90)	1742.25 (±398.70)	1378.29 (±359.62)	1042.94 (±603.93)

Annotations. Means and standard deviations in parentheses are reported.

Supplementary Table S7. *Full model calculation with affectedness by restrictions.*

**index**	**β**	**SE**	**95%-CI**
sex	57.14	48.07	-38.79; 153.44
age	-4.49	1.58	-7.62; -1.36
T2	43.51	26.38	-8.60; 96.12
T3	97.66	29.47	39.29; 155.99
affectedness	1.05	8.25	-15.46; 17.35
age x T2	-2.34	1.57	-5.47; 0.77
age x T3	-4.23	1.62	-7.44; -1.01
age x affectedness	0.02	0.51	-1.00; 1.04
T2 x affectedness	-13.60	9.72	-32.81; 5.66
T3 x affectedness	12.17	11.13	-9.80; 34.31
age x T2 x affectedness	-0.34	0.62	-1.56; 0.88
age x T3 x affectedness	-1.17	0.63	-2.43; 0.08

Supplementary Table S8. *Full model calculation with depressiveness by restrictions.*

index	β	SE	95%-CI
sex	77.36	48.51	-19.73; 174.11
age	-7.56	4.29	-16.04; 0.91
T2	-30.23	81.41	-191.01; 132.00
T3	123.38	77.21	-29.43; 276.44
depressiveness	1.15	27.27	-52.72; 55.21
age x T2	-5.93	4.64	-15.12; 3.26
age x T3	-8.63	4.48	-17.53; 0.23
age x depressiveness	1.20	1.61	-1.98; 4.40
T2 x depressiveness	34.47	31.85	-28.84; 97.41
T3 x depressiveness	-19.60	30.52	-80.06; 40.84
age x T2 x depressiveness	1.96	1.84	-1.69; 5.60
age x T3 x depressiveness	2.62	1.79	-0.92; 6.17

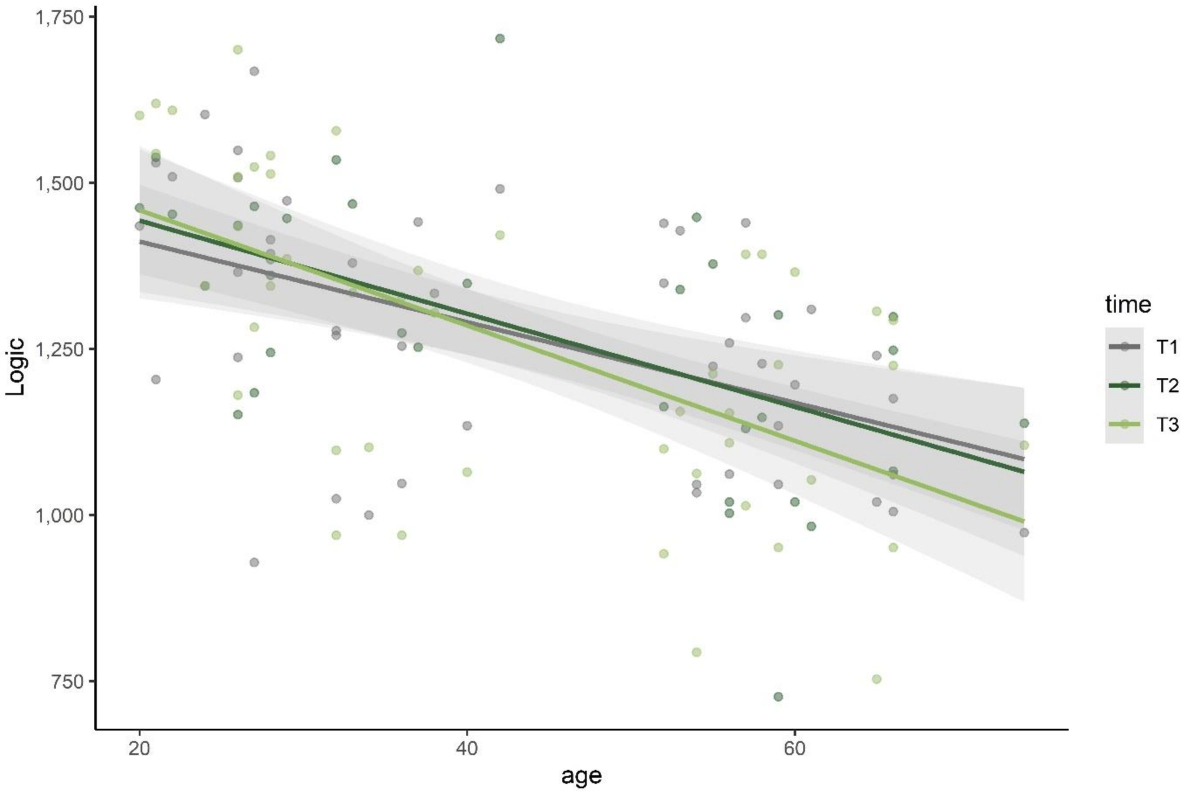

Supplementary Figure S9. Age-related logic task performance in the three assessments T1 to T3. Shaded areas represent 95% confidence interval.

Supplementary Table S10. *Full model working memory with depressiveness.*

index	β	SE	95%-CI
sex	21.31	54.36	-87.22; 130.02
age	-11.09	6.51	-23.96; 1.80
T2	85.96	138.93	-190.08; 360.56
T3	23.89	124.38	-222.65; 270.01
depressiveness	-34.10	48.15	-129.17; 61.04
age x T2	-16.87	7.63	-31.98; -1.74
age x T3	-16.55	7.07	-30.61; -2.57
age x depressiveness	0.82	2.68	-4.50; 6.12
depressiveness x T2	31.78	59.78	-86.34; 150.65
depressiveness x T3	50.84	51.15	-50.34; 152.26
age x T2 x depressiveness	6.30	3.32	-0.28; 12.88
age x T3 x depressiveness	5.85	2.91	0.10; 11.62

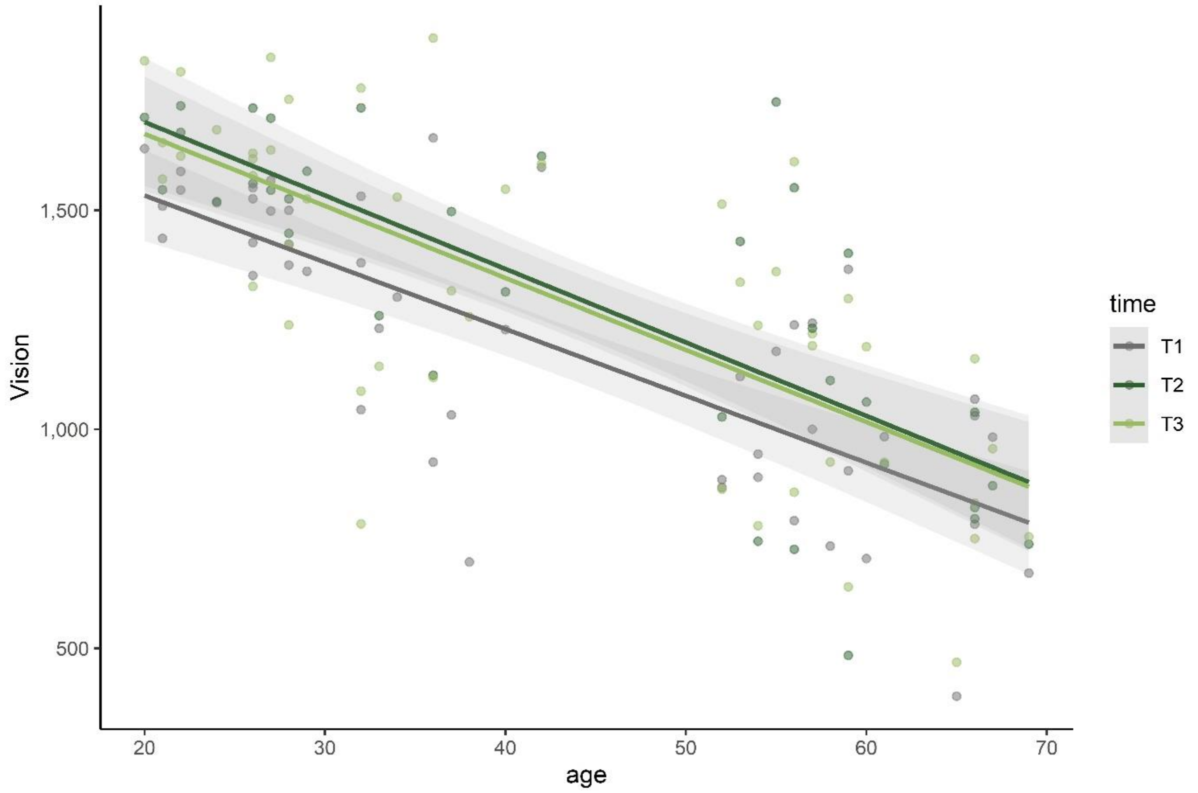

Supplementary Figure S11. Age-related vision task performance in the three assessments T1 to T3. Shaded areas represent 95% confidence interval.

Supplementary Table S12. *Items of the subjective cognitive scale. Participants were asked to rate how well or poorly they master the listed skill. Answers were given on a visual analog scale ranging from 0 (very poorly) to 100 (very well). The instruction is specified at the top. The questionnaire, from which certain items are derived is given in italic (EMQ = Everyday Memory Questionnaire; DEX = Dysexecutive Questionnaire; FEDA = Questionnaire for Experiences of Attention Deficits).*

Questioned ability	Dimension
In the following you will find a list of skills, that relate to mental performance. Please use the slider to rate how well or how poorly you master the skills. In some cases, examples are given in parentheses for better illustration.	
1. Remembering memories of important moments of my life (friends from kindergarten, marriage, first kiss)	Explicit long-term memory
2. Remembering birthday of close friends or relatives	Explicit long-term memory
3. Learning of new movements (dancing, riding bicycle)	Implicit memory<br>*adapted from EMQ*
4. Self-discipline (only buying things that are needed)	Executive functions<br>*adapted from DEX*
5. Emotional control (not raising voice in argument)	Executive functions<br>*adapted from DEX*
6. Coping with negative feelings by looking at the situation from a different perspective	Executive functions
7. Don’t let little things upset me	Executive functions<br>*adapted from DEX*
8. Plan and coordinate actions to reach a certain goal (errands for a birthday party, planning to move)	Executive functions<br>*adapted from DEX*
9. Sort tasks by importance and work through them if it is not possible to complete them all directly	Executive functions<br>*adapted from DEX*
10. Complete tasks as they occur and do not put them off until later	Executive functions<br>*adapted from FEDA*
11. Flexibility in case of spontaneous changes (bypassing a construction site on my usual way to the supermarket)	Executive functions
12. Following the plot line while reading a book	Working memory<br>*adapted from FEDA/EMQ*

13. Remembering a telephone number after it was told to me a few seconds before	Working memory *adapted from EMQ*
14. Focusing on several things at once (cooking while having a conversation)	Attention
15. Maintaining concentration for a long period of time (during long car rides)	Attention *adapted from FEDA*
16. Ignoring distracting things (ignoring the buzzing refrigerator while reading a book)	Attention *adapted from FEDA*
